# Rehabilitation in the context of WHO’s mandate

**DOI:** 10.2471/BLT.22.289236

**Published:** 2022-11-01

**Authors:** Tedros Adhanom Ghebreyesus

**Affiliations:** a World Health Organization, Avenue Appia 20, 1211 Geneva 27, Switzerland.

Rehabilitation is the interventions needed when a person is experiencing limitations in everyday physical, mental and social functioning due to ageing or a health condition, including noncommunicable diseases or disorders, injuries or trauma.^1^ Rehabilitation helps a child, adult or older person to be as independent as possible in everyday activities and enables participation in education, work, recreation and family life. Rehabilitation does so by addressing underlying conditions; by helping to overcome difficulties with thinking, seeing, hearing, communicating, eating or mobility; and by providing, adapting and training in the use of assistive products.

Today, an estimated 2.4 billion people live with conditions that could benefit from rehabilitation. This number has increased by about two thirds during the past three decades and reflects the enormous and growing need for rehabilitation. However, many people do not receive the rehabilitation they require, especially in low- and middle-income countries, where more than half of those who need rehabilitation do not receive it.^2^

The main drivers of this global need are ageing, the increased prevalence of noncommunicable diseases, and people living with the consequences of injuries. However, as shown by recent events such as the coronavirus disease 2019 pandemic and conflicts around the world, infectious diseases and health emergencies can also add significantly to the demand for rehabilitation.

The seriousness of this global health challenge cannot be overstated. In 2017, the World Health Organization (WHO) launched the Rehabilitation 2030 initiative, a call to action to rally stakeholders to strengthen health systems for rehabilitation.^3^ To date, WHO has supported over 20 countries around the world to strengthen their health systems so they can better provide rehabilitation services.

To boost action at the country level, WHO has developed technical tools to support the integration of rehabilitation into national health plans and across various areas of health – including noncommunicable diseases, mental health and ageing – as well as into health emergency responses and primary care.^4^ These tools are now being used by WHO, health ministries and development partners around the world. In addition, a strong global narrative underscoring rehabilitation as an essential health service and part of country efforts towards achieving universal health coverage has been established. To maintain momentum, WHO recently launched the World Rehabilitation Alliance, a global network of stakeholders to conduct evidence-based advocacy to raise awareness of and demand for rehabilitation, as well as for networking, knowledge-sharing and promoting the narrative around rehabilitation.^5^

As part of its commitment to rehabilitation and more generally to science, WHO also called for papers on health policy and systems research to assist policy-makers in their decisions around issues such as the financing and governance of rehabilitation, as well as implementing and monitoring these decisions.^6^ Such research can help to guide decisions on policies and actions to support and invest in rehabilitation. This theme issue of the *Bulletin of the World Health Organization* contains examples of the work of multidisciplinary teams across the world who have come together to understand the need for and potential of integrating rehabilitation into specific disease areas, as well as along the continuum of care and in primary care.

Together with WHO, the rehabilitation community is committed to strengthening evidence-based health systems for rehabilitation. The call now is for the global public health community to also join the journey, and for those working in the health sector to understand how societies and health systems can enable people not only to live longer but also to live better through making rehabilitation services available to those who need them. My hope is that all those involved in public health will recognize rehabilitation as part of their core business. Ultimately, rehabilitation is an essential component of Health for All.
